# GIS-based multi-criteria decision models for barite exploration in Nigeria’s Benue Trough

**DOI:** 10.1038/s41598-024-63996-8

**Published:** 2024-06-18

**Authors:** Jiriko N. Gajere, Olabisi A. Adekeye, Andongma W. Tende, Mohammed D. Aminu

**Affiliations:** 1 Department of Geology and Mining, University of Abuja, Abuja, Nigeria; 2 Department of Geology and Mineral Sciences, University of Ilorin, Ilorin, Nigeria; 3 Department of Geology, Kano University of Science and Technology, Wudil, Nigeria; 4 Clean Air Task Force, Boston, MA 02109 USA; 5 Department of Geology, University of the Free State, Bloemfontein, South Africa

**Keywords:** Economic geology, Geology

## Abstract

Spatial predictive mapping using geographic information system (GIS) is considered an invaluable tool for reconnaissance-scale exploration of mineral resources. In this study, geospatial data on geophysics, remote sensing, and structural and lithological attributes were systematically integrated to prospect barite potential zones within the Mid-Nigerian Benue Trough (MBT). Correlation attribute evaluation was used to establish the relationship between mineral deposit occurrences and geospatial data, while data integration was implemented using the Multi-Objective Optimization by Ratio Analysis (MOORA), Technique for Order of Preference by Similarity to Ideal Solution (TOPSIS), and Additive Ratio Assessment (ARAS) multi-criteria models. Here we show that the correlation attribute evaluation suggests that barite occurrences displayed a strong correlation with spatial data on lineament density, ferric iron alteration, and potassium to thorium (K/Th) ratio, whereas a weak correlation was observed with spatial data on the first vertical derivative (FVD), proximity to the host rock, and ferrous iron alteration. Here we report that the quantitative estimation of predictive models indicated that very high predictive zones for barite occurrences accounted for 19% of all the models. The accuracy assessment using Receiver Operating Characteristic (ROC)/Area Under the Curve (AUC) showed prediction levels above 78% for all models. The effectiveness of the spatial application of multi-criteria decision models makes them a reliable tool for barite exploration within the Mid-Nigerian Benue Trough (MBT) and other geologically similar environments.

## Introduction

Mineral exploration is a challenging task and requires a systematic approach for in-depth analysis to identify all locations associated with the economic concentration of a given deposit type. On a regional scale, there is a compelling need to approach exploration exercises using all the available information. In most cases, a combination of existing geoscientific data such as geological, geophysical, geochemical, structural, and satellite data are indispensable requirements for an effective and thorough investigation of mineral resource occurrences^1,2^. Remote sensing (RS) and GIS-based techniques are a reliable platform for the integration of all these pools of geodata^3,4^. Sabins^5^ described RS as the science of acquiring, processing, and interpreting images and related data, acquired from aircraft and satellites that record the interaction between matter and electromagnetic energy. The MBT has long been a hub for barite mineralization, and some work has been done in the study area. Yet, there exists no thorough, coordinated, integrated, and up-to-date mineral exploration data on barite potential in the study area. The Mid-Nigerian Benue Trough (MBT) which has a suitable geological setting for barite mineral potential, has datasets that could aid far-reaching mineral exploration programmes^6^. These datasets, if well-harnessed and integrated, can effectively attract substantial investments to the less impactful Nigerian mineral and mining industry. Unfortunately, the absence of up-to-date mineral potential maps has regrettably kept down the much-needed investment in the MBT and, by extension, the mineral and mining sector of Nigeria.

Geological investigations of mineral deposit occurrences involve the deployment of several methods. These methods include geophysics, geochemistry, petrology, mineralogy, and RS/GIS for mineral potential assessment and target delineation of mineral resources within different geological terrains and environments^7–11^. Primarily, geophysical, and geochemical methods have been extensively applied for mineral exploration exercises^12^. However, current advances in RS/GIS methodologies in concurrence with other traditional exploration methods across numerous mineral belts around the world have been vital for discovering a lot of mineral occurrences^13–15^.

Carranza and Sadeghi^14^ argue that applying RS and GIS on a smaller mapping scale is the most efficient option. This has been attributed to its effectiveness, prediction accuracy, and ability to explore inaccessible terrains, leading to significant achievements in mineral exploration. Carranza and Hale^16^ reported that the traditional geological mapping techniques and mineral exploration methods are comparably costly, involve considerable capital outlay, and require longer periods of investigation. Meanwhile, more recent exploration techniques, such as RS and GIS, are cost-effective, fast, and possess an enabling platform for digital data integration and modelling with great efficiency^17^. The three main aspects of the applications of RS/GIS techniques to mineral exploration are often anchored on the structural investigation of ore geometry, satellite mapping of hydrothermal alteration, and spatial data integration for mineral potential map development^18,19^. The spatial data integration of geological datasets is crucial amongst all other methods because it is incorporative, inclusive, extensive, and a unifier of all other geological data from various sources.

The application of data integration techniques is commonly centered on the availability of suitable mineral deposit data. Typical of most underdeveloped and developing countries lacking technological advancements, the deployment of RS and GIS technology in the search for mineral deposits in Nigeria which has high prospects of varieties of mineral occurrences is far from satisfactory^12^. Apart from the limitations related to technological advancement, other characteristic inhibitions include inaccessible terrains and the excessive cost of exploration exercises. The application of RS and GIS has been proven to be an effective remedy as it has been adjudged as the most proficient option and is ascribed with enormous achievements concerning mineral exploration^12,20^.

The capability of RS and GIS techniques to map and delineate hydrothermal alteration zones and geological structures linked to mineral occurrences has made it the most appropriate technique^11^. Furthermore, geophysical datasets in the form of aeromagnetic or radiometric data can be processed to extract valuable mineral deposit signatures^21,22^. This study applies optimized knowledge-driven techniques such as TOPSIS, MOORA, and ARAS to predict zones favorable to barite mineralization within the MBT. These three methods are considerably new, although TOPSIS was applied to mineral exploration investigations earlier than the application of ARAS and MOORA techniques^23^.

## Geological setting

**Figure 1:**
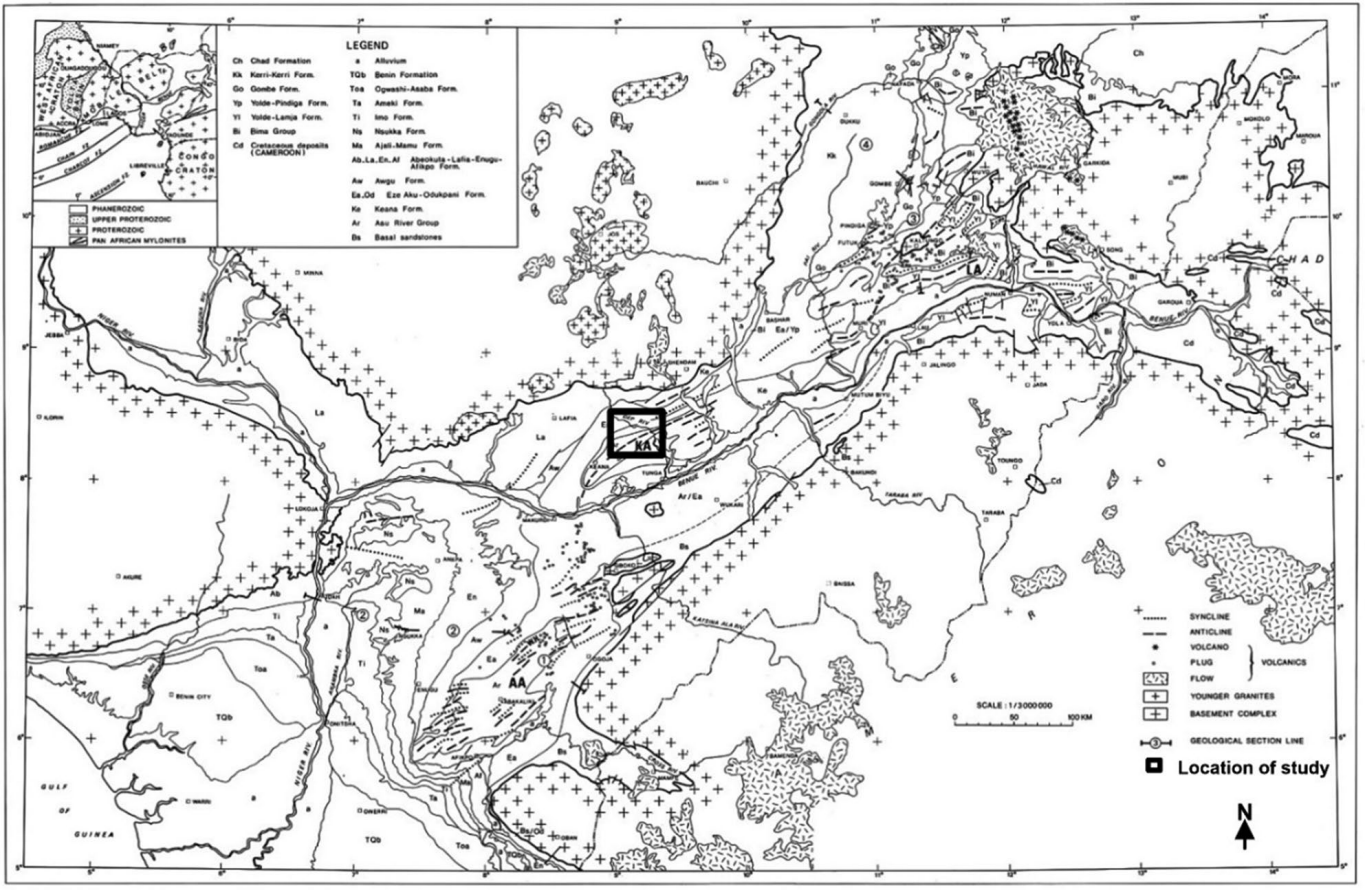
Regional geology map of the Benue Trough showing the study location (Mid-Nigerian Benue Trough)^3^. Map was modified after Benkhelil^27^.

**Figure 2:**
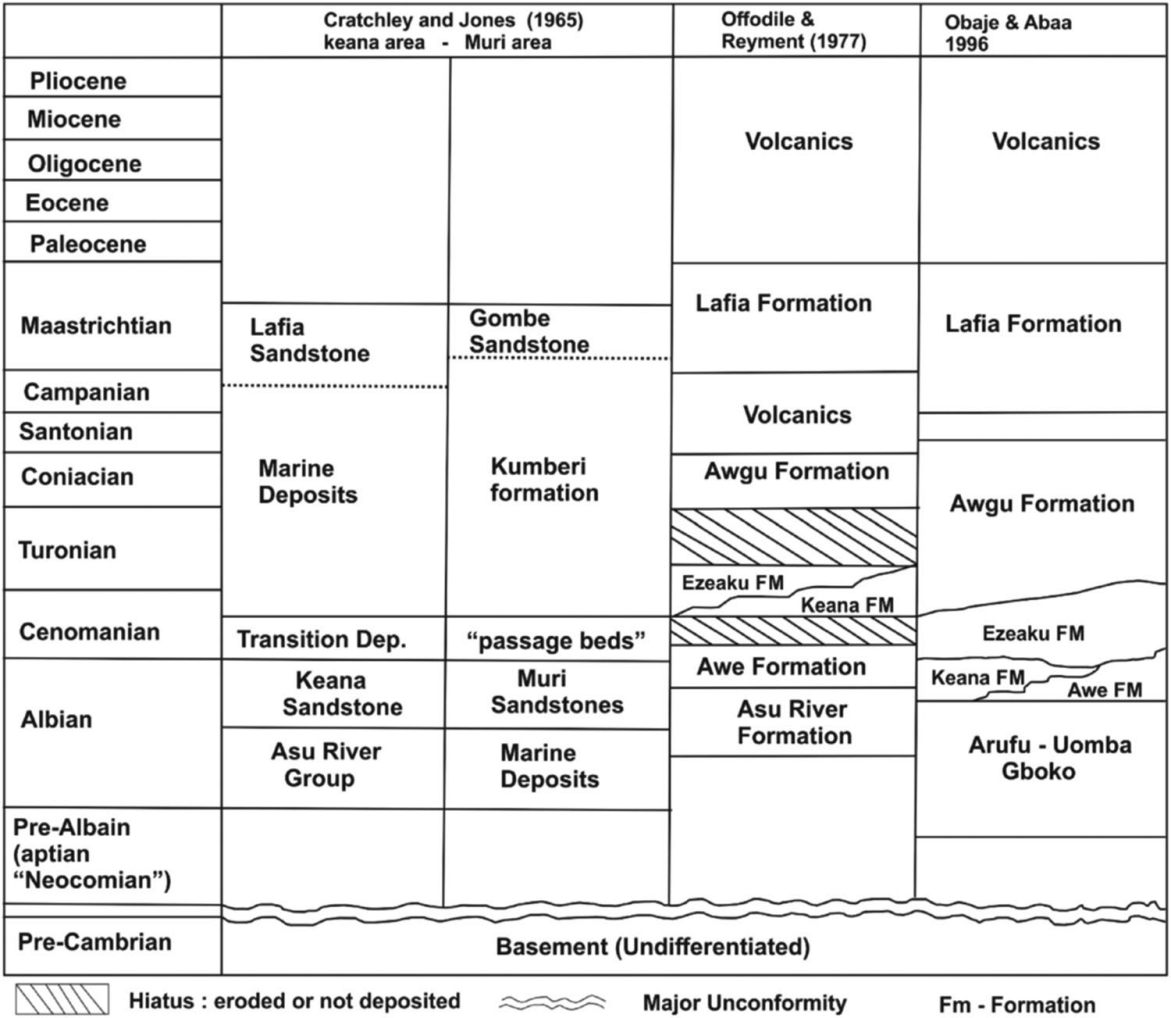
The Stratigraphic succession in the Mid-Benue Trough^30^.

The Nigerian Benue Trough (Fig. 1), as described by Offodile^24^ and Ofoegbu^25^, is an 80–90 km wide fault-bounded depression containing up to 5000 m thick deformed Cretaceous sedimentary and volcanic rocks. It is an intracontinental sedimentary basin in which most of the sediments have been folded and faulted because of later isostatic readjustment along faults in the underlying basement^26^. The Benue Trough (BT), formed during the Cretaceous because of the opening of the Atlantic Ocean, was described by Benkhelil^27^ as an intracontinental basin approximately 1000 km long and 100–150 km wide. It rests unconformably on the Precambrian basement complex and stretches in the NE-SW direction. The trough extends from the southern limit of the Chad Basin to the northern limit of the Niger Delta. Among the several models postulated on how the BT was formed, the few most recently proposed models are inclined toward a pull-apart system in which wrenching is considered a principal tectonic process leading to the evolution of the trough. The BT was arbitrarily segmented into the lower, middle, and upper portions with diverse stratigraphic sequences (Fig. 2). No actual line of the boundary can be drawn to demarcate the specific portions, but key localities (towns/settlements) that establish the depocenter of the various sections have been well recognised and documented^26,28,29^.

### The Mid-Nigerian Benue Trough

The study location (Fig. 1) is an integral part of the Nigerian BT. The MBT has an estimated content of Cretaceous-Tertiary sediment content of up to 6 km thick. Offodile^31^, and Offodile and Reyment^32^ described the geology of the MBT as consisting of six lithostratigraphic formations comprising the Asu River group, Awe, Keana, Eze-Aku, Awgu, and Lafia Formations (Fig. 2). The oldest sediments which are the Asu River group dated between mid and late Albian, were a result of the marine transgression of the South Atlantic and the Gulf of Guinea. The lithological components of the Albian marine Asu River group are composed of shales, calcareous shales, limestone, micaceous siltstones, and mudstones^8^. Other earlier studies by Peters^28^ and Offodile and Reyment^32^ reported that the Asu River group was fossiliferous with abundant ammonites, agglutinated, and foraminiferal taxa. Offodile and Reyment^32^ described localities around Awe and east of Keana, as well as the crest of the Keana anticline, as some of the most conspicuous outcrops seen within this study location.

The older Asu River group Formation is overlain by transitional beds of the Awe Formation which consists of flaggy, whitish, and medium-to coarse-grained calcareous sandstones, siltstones, carbonaceous shales, and clays which Obaje^8^ and Obaje et al.^33^ considered as a passage or transitional beds. Offodile and Reyment^32^ reported an estimate of 100 m for the Awe Formation in the Awe town and towards Abuni. One of the most identifiable characteristics of the sandstone units of the Awe Formation as reported by Offodile ^24^ is the presence of incessant coarsening upward and the decreasing micaceous content towards the top of the formation. The Awe Formation represents a late Albian to early Cenomanian regression that has been traced over a large extent of the BT.

The Cenomanian regressive phase produced the Keana Formation which overlies the Awe Formation and comprises basically of cross-bedded fine to very coarse-grained which Obaje^8^ and Obaje et al.^33^ considered as conglomerates of gritty/arkose sandstones of fluvial-deltaic origin. Outcrops of the Keana Formation predominate within and around the eastern and western flanks of the Keana anticline, where the thickness is estimated to be 800 m as reported by Ofoegbu^25^ and Offodile^31^. The Eze-Aku Formation which overlies the Keana Formation was reported by Obaje et al.^34^ as a product of the late marine transgression that ensued during the Cenomanian. Obaje et al.^34^ described the Eze-Aku Formation as greyish-to-black shales with clay horizons containing fine-to-medium-grained sandstones and limestone intercalation. The Eze-Aku Formation inter-fingers with the Awgu Formation at its southern boundary. The Awgu Formation which signified a sudden conclusion of marine sedimentation in the MBT overlays the Eze-Aku Formation^33^. The continuous presence of shaley limestone, calcareous and carbonaceous shales, and coal beds within the Awgu Formation suggests an abrupt end of marine sedimentation in the trough.

The Lafia Formation consists of Maastrichtian, poorly consolidated continental ferruginous sandstones (mostly cross-bedded) that occur together with flaggy mudstones and claystone containing a paleosol horizon. This formation is the youngest sedimentary depositional phase in the MBT and the entire BT^24^. Obaje et al.^33^ reported that the characteristic ferruginous nature of the Lafia Formation is suggestive of a syn-depositional origin aided by strong oxidizing conditions as the major catalytic feature. The Mid-Santonian was a time of folding throughout the BT^34^ and the post-folding Campano-Maastrichtian Lafia Formation, deposited under continental circumstances terminated the sedimentation regime in the BT, after which broad volcanic emplacements took control in the Tertiary^24^. Major barite mineralization is thought to be affiliated with the mineralized hydrothermally altered veins, which are considered to be a result of tectonic rifting that led to the emplacement of the BT^27^. These faults are also thought to have been responsible for creating pathways through which hydrothermal veins of possibly magmatic origin^35^ or remobilized meteoric waters enriched in Ba percolated.

### Barite mineralization in Nigeria

Mineral barite which usually occurs primarily as gangue in gangue in several metallic ore deposits, is an essential industrial mineral with a wide variety of applications. The existence of barite mineralization in igneous, metamorphic, and sedimentary lithologies dated from the Early Archean to Late Phanerozoic, as reported by Hanor^36^, shows its flexibility in diverse geological environments. The mode of occurrence of barite deposits is primarily bedded or stratiform, as residual deposits, and in vein and cavity fillings. Most of the barite mineralization within the BT occurs as vein/cavity filling^37–39^.

Several locations in Nigeria have been reported to have occurrences of barite deposits mainly within the NE-SW trending Cretaceous BT^39–41^. Maurin and Benkhelil^42^, reported that the distribution of barite deposits and other deposits such as Pb–Zn–Cu–F mineralization was structurally controlled and attributed same to the opening/rifting of the trough. There has been intense debate regarding the origin of barite mineralization in the BT with the source of the metal barium, and the source of the mineralizing fluids as the key contentious point. For example, Omada and Ike^43^ and Ezepue^44^ favored the magmatic-hydrothermal fluid resulting from the Tertiary volcanic as a likely source of the mineralizing fluid while the consideration of both igneous and sedimentary rocks as potential sources of the mineralizing fluid was reported by Orajaka^45^. Olade and Morton^46^ proposed the circulating brine model, which is based on what was described as the high geothermal gradient associated with the rifting of the BT. Akande et al.^40^ proposed a basinal brine expulsion model established from fluid inclusion and isotope studies of barites in the lower and middle BT. As stated earlier, the source of metal (Ba) is still a subject of controversy, notwithstanding the numerous studies conducted for example Olade and Morton^46^ and Akande et al.^40^ to establish the source of ore constituents^39,47^.

## Materials

The target data for barite mineralisation include the Global Positioning System (GPS) readings of the major barite mines across the study location. Preliminary information on this data was obtained from the existing literature to ascertain possible occurrences within the MBT. A field survey was conducted to map and record all possible barite mines across the study area. The Garmin eTrex® 10 receiver was used to obtain the geographic coordinates of existing mines and upon return from field studies, the geographical coordinates were entered into an Excel sheet where they were converted into point data using ArcMap software. The existing point data were then used to ascertain the reliability of each spatial dataset and predict the accuracy levels for each predictive model.

Predictive mapping of barite mineralization within the study location was developed using spatial data from geophysics, satellite imagery, and lithological and structural information. Table 1 encapsulates all the spatial data used in the development of mineral predictive maps. Spatial geophysical data include aeromagnetic and aero-radiometric data obtained from the Nigerian Geological Survey Agency (NGSA). The magnetic data for the study location, consisting of Total Magnetic Intensity (TMI) data, constitutes part of a regional magnetic survey acquired for the NGSA by Fugro Airborne Surveys Ltd, at a flight height of 80 m on a series of NW–SE flight lines. The magnetic data were digitally filtered by applying the FVD algorithm to generate magnetic evidential data. Spatial radiometric data were obtained from the NGSA as grid multichannel data of potassium (K), thorium (Th), and uranium (U). Radiometric data were obtained along the NW–SE flight line at an average elevation of 100 m, flight spacing of 500 m, and tie lines at 200 m in the NE-SW direction. Spatial data on ferric/ferrous iron alterations and host rock were obtained from well-processed Landsat imagery downloaded from the Earth Explorer website (www.earthexplorer.usgs.gov).

**Table 1:** Summary of data sources and spatial data applied in mineral predictive analysis.

Sn	Data type	Source	Primary data type	Processed data type
1	Geophysical data	NGSA	Aeromagnetic	FVD
2	Geophysical data	NGSA	Aero-radiometric	K/Th ratio
3	Iron alteration data	www.earthexplorer.usgs.gov	Landsat ETM	Ferrous iron alteration
4	Iron alteration data	www.earthexplorer.usgs.gov	Landsat ETM	Ferric iron alteration
5	Lithological data	www.earthexplorer.usgs.gov	Landsat ETM	Distances to host rock (DHR)
6	Structural data	www.earthexplorer.usgs.gov	DEM	Lineament density

The barite mineralization evidential themes applied in this study include geological, geophysical, and structural data. This was done by integrating remote sensing (RS) and geographic information system (GIS) techniques to gain a comprehensive understanding of the barite mineral potential in the area. The application of aeromagnetic data was to identify magnetic anomalies associated with geological structures favorable to barite mineralization, while airborne radiometric data was used to highlight variations in the ratio of radioactive elements linked to barite mineral potential. Aeromagnetic and airborne radiometric methods were preferred due to their ability to cover large areas efficiently and at relatively low costs. This was particularly important as the MBT is a difficult terrain. They also complemented other exploration techniques, providing valuable geological information. However, these techniques are sensitive to environmental factors and may be affected by cultural or anthropogenic interference, leading to inaccuracies in data interpretation. To remedy these limitations, other exploration methods such as field mapping and advanced data processing techniques were applied. These measures helped to identify and delineate prospective areas for barite mineralization within the MBT.

The Landsat ETM data consists of eight bands, including the visible (2), near-infrared (2), short-wave infrared (2), thermal infrared (1), and panchromatic band (1). The distribution of ferrous and ferric iron alteration in the study location was obtained by digitally processing Landsat imagery using the band ratio. The ferrous iron distribution was obtained by dividing band 5 and band 4, while the ferric iron alteration was generated by dividing band 3 on band 1^18^. Band rationing is a widely used technique in remote sensing and geological studies due to its ability to extract more information, differentiate between spectral signatures, reduce noise, and discriminate between geological units. This technique also facilitates dimensionality reduction and supports exploratory data analysis, which ultimately leads to more effective mapping of barite mineral potential.

Information on lithological distribution was obtained from the digital processing of Landsat data using the principal component analysis. A false color composite imagery generated from the first three principal components enhanced lithological variations across the study area. Lithological units hosting barite mineralization were extracted and subjected to buffering as a means of generating rasterized distances to existing host rock^20^. Regional structural data designated as lineaments were extracted from a high-resolution digital elevation model (DEM) using manual digitisation^48^. The DEM data was downloaded from the Earth Explorer website, www.earthexplorer.usgs.gov. The extracted lineaments were then subjected to the kennel density algorithm generating lineament density for the study location. The lineament density was generated by evaluating the number of lineaments per unit area^49^.

### Methods

**Figure 3:**
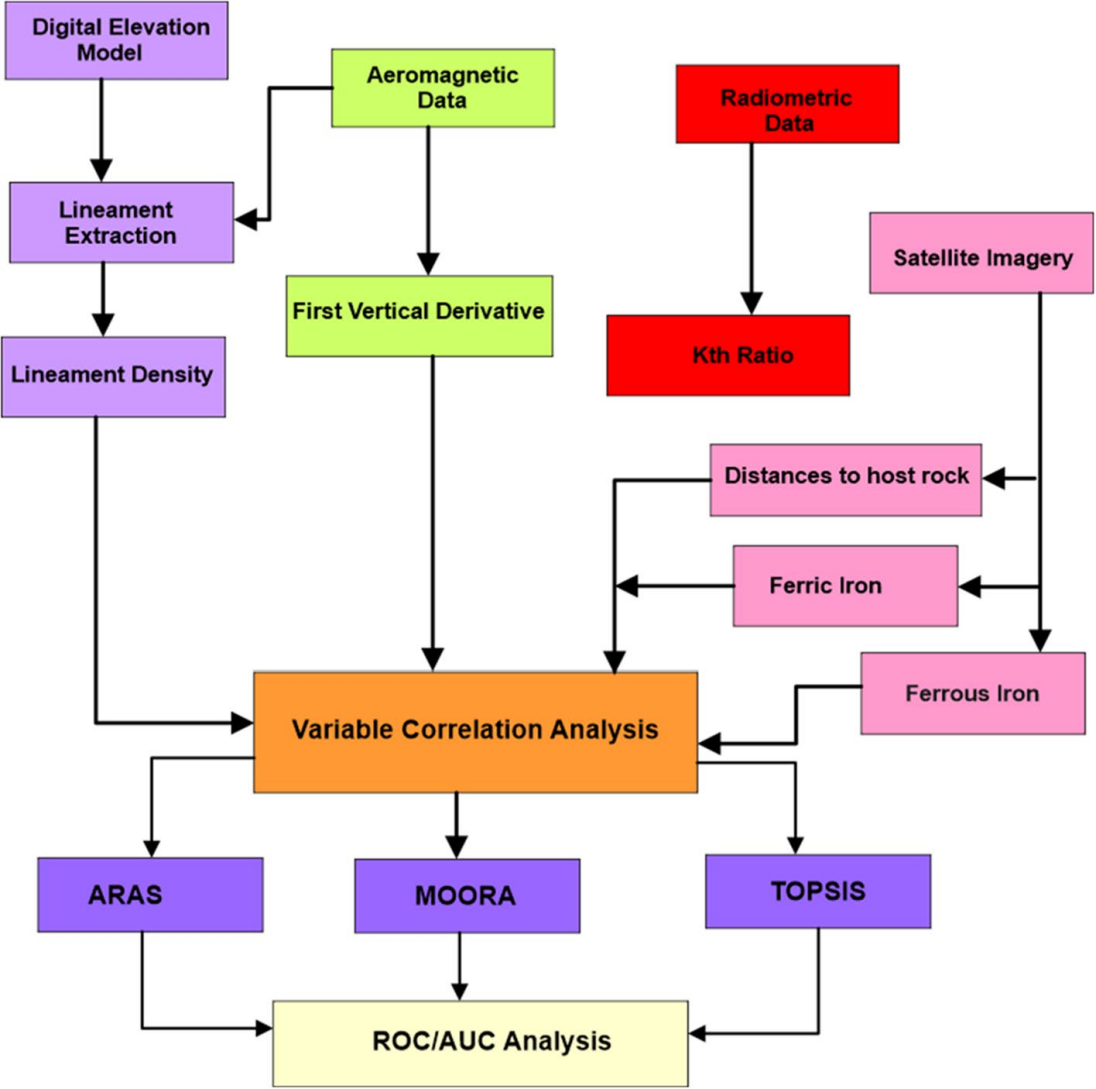
A summarised flowchart for the methods used in the study.

A flowchart illustrating the methods applied in this study is shown in Fig. 3. Various parameters and their interrelationships are described in a spatial illustration.

### Evaluation tools

#### Evaluation of correlation attributes

Attribute evaluation procedures are generally considered vital tools in predictive mapping because they permit concise visualisation and promote an in-depth understanding of spatial data structure by identifying the most relevant data for predictive mapping. Generally, these approaches play an important role in the dimensionality of data by eliminating the most irrelevant attributes in each dataset^50^. Correlation attribute evaluation (CAE) employs a correlation-based heuristic to evaluate the relative importance of attributes within a given model. Generally, it considers the usefulness of individual attributes for predicting the class label along with their level of intercorrelation^51^. Statistically, CAE can be calculated using Eq. 1.

1 $${r}_{zc}=\frac{k{\overline{r} }_{zi}}{\sqrt{k+k(k-1){r}_{ii}}}$$

where:

${r}_{zc}$ represents the correlation or dependence between the features and the class variables, while $k$ is the number of features, ${\overline{r} }_{zi}$ is the average correlation between the feature class and ${r}_{ii}$ is the average intercorrelation between a feature to another feature.

#### Receiver operating characteristic curve/area under curve

The confidence level obtained from the performance evaluation of every GIS model is vital to its application particularly to the search for mineral deposits occurrences as it gives credence to the results^52^. When dealing with multiple threshold values, the ROC analysis and AUC are considered a more vigorous approach for appraising the accuracy and confidence level of GIS predictive models because they demonstrate an independent effect on those multiple threshold values^53^. The ROC can be statistically implemented by plotting the sensitivity against 1-sensitivity values in a binary curve. Equations 2 and 3 demonstrate a statistical formula for computing the sensitivity and specificity of a given prediction.

2 $$SN=\frac{TP}{TP+FN}$$

3 $$SP=\frac{TN}{TN+FP}$$

From these equations, SN is the sensitivity, SP represents the specificity. TP, TN, FP, and FN are the true positive, true negative, false positive, and false negative.

In applying AUC in model evaluation, the efficiency is usually ranked between 0–1. It evaluates the performance of every GIS model via a binary classification system along with a continuous variable 64. In the assessment of a GIS model using AUC, the closer the values are to 1, the better the performance of the applied model, whereas values closer to 0 indicate poor performance of the predictive model^52^. The study applied both the AUC and ROC techniques in the qualitative and quantitative evaluation of the knowledge-driven predictive models for barite mineralisation in the Cretaceous MBT.

## Methods

### MOORA

MOORA is a multi-criteria decision-making technique used to enhance the most suitable alternatives and find the most feasible substitute within a set of options^54^. Raju et al.^55^ reported that MOORA to a greater extent accomplishes a top-level degree of effectiveness in the rating and choice of criteria, as it is effectively free from intricacies. This optimal selection ability of MOORA has been attributed to its ability to recognise and distinguish several contradictory criteria into positive (optimal) and negative (minimal) attributes^56^. In the process of identifying and segregating various conflicting criteria, unsuitable criteria were eliminated while strengthening the selection process. The MOORA predictive process is most often statistically implemented in steps, as highlighted in the four (4) steps.

*Step 1* generate a decision matrix of responses to different alternatives on objectives.

4 $$X = \left[ {\begin{array}{*{20}l} {X_{11} } \hfill & \cdots \hfill & {X_{1i} } \hfill & \cdots \hfill & {X_{1n} } \hfill \\ \vdots \hfill & \ddots \hfill & \vdots \hfill & {\mathinner{\mkern2mu\raise1pt\hbox{.}\mkern2mu \raise4pt\hbox{.}\mkern2mu\raise7pt\hbox{.}\mkern1mu}} \hfill & \vdots \hfill \\ {X_{j1} } \hfill & \cdots \hfill & {X_{ji} } \hfill & \cdots \hfill & {X_{jn} } \hfill \\ \vdots \hfill & {\mathinner{\mkern2mu\raise1pt\hbox{.}\mkern2mu \raise4pt\hbox{.}\mkern2mu\raise7pt\hbox{.}\mkern1mu}} \hfill & \vdots \hfill & \ddots \hfill & \vdots \hfill \\ {X_{m1} } \hfill & \cdots \hfill & {X_{mi} } \hfill & \cdots \hfill & {X_{mn} } \hfill \\ \end{array} } \right]$$

where ${X}_{ij}$—the response of the alternative $j$ on the objective or attribute $i$*;* $i = 1,2, \ldots ,n$—is the number of objectives or attributes; ${\text{j }} = { }1,{ }2, \ldots ,{\text{m}}$—is the number of alternatives.

*Step 2* Following the first step, the decision matrix is normalised using the formula in Eq. (5).

5 $${x}_{ij}^{*}= \frac{{x}_{ij}}{\sqrt{\sum_{j=1,}^{m}{x}_{ij}^{2}}}$$

where $${x}_{ij}$$—response of alternative $$j$$ on objective $$i$$; $$j = 1,2,3, \ldots ,m$$$$;$$ $$m$$—the number of alternatives; $$i = 1,2, \ldots ,n$$$$;$$ $$n$$—the number of objectives; $${x}_{ij}^{*}$$—a dimensionless number representing the normalized response of the alternative $$j$$ on the objective $$i$$.

*Step 3* Step three involves the enhancement of different attributes. This is realised using the application of Eq. (6), whereby the normalised performance in the case of beneficial attributes (maximisation) and deduction in the case of non-beneficial attributes (minimisation) are added. The optimisation process of the different criteria is employed in Eq. (6).

6 $${y}_{j}^{*}= \sum_{i=1}^{i=g}{x}_{ij}^{*}- \sum_{i=g+1}^{i=n}{x}_{ij}^{*},$$

where $i=1, 2, \ldots ,g$—the objectives to be maximized;

$i = g + 1,g + 2, \ldots ,n$—the objectives to be minimized;

${y}_{j}^{*}$—the normalized assessment of alternative $j$ with respect to all objectives.

An ordinal ranking of ${y}_{j}^{*}$ shows the final preference.

*Step 4* The weight factor is introduced in Eq. (7) because the attributes have different degrees of relevance in the prediction process.

7 $${y}_{j}^{*}=\sum_{i=1}^{i=g}{{W}_{j}*x}_{ij}^{*}- \sum_{i=g+1}^{i=n}{{W}_{j}*x}_{ij}^{*},$$

${W}_{j}$ represents the weight of the ${j}^{th}$ attribute.

Depending on its favourable attribute (maximal) and unfavourable attribute (minimal) number, the resultant value reflected in ${y}_{j}^{*}$ may either be positive or negative. The order of the ranking of ${y}_{j}^{*}$ indicates its resultant preference.

### TOPSIS

The TOPSIS model is a multi-criteria evaluation technique that has been widely applied in the predictive mapping of mineral occurrences^57,58^. The model was originally developed by Hwang and Yoon^59^ based on the fundamental principle of creating the least distance between all possibilities and the optimal (positive) ideal solution while maintaining the highest distance to the minimum (negative) ideal solution. In general, the minimum (negative) ideal solution makes up most of the cost criteria at the expense of minimising cost benefits. TOPSIS is typically related to a simple knowledge-driven prospective model which has a comparative advantage, especially when handling exploration complications associated with a substantial number of alternatives^60^. Statistically, according to Dagdeviren et al.^61^, the TOPSIS predictive model is systematically implemented in a six-step procedure.

*Step 1* A decision matrix was established for the ranking. The structure of the decision matrix can be expressed as follows.

8 $$\begin{gathered} \quad\quad\quad\quad \;F_{1} \;\;F_{2} \;\; \cdots \;\;F_{j} \;\; \cdots \;\;F_{n} \hfill \\ D = \begin{array}{*{20}l} {A_{1} } \hfill \\ {A_{2} } \hfill \\ { \vdots } \hfill \\ {A_{i} } \hfill \\ \vdots \hfill \\ {A_{J} } \hfill \\ \end{array} \left[ {\begin{array}{*{20}l} {f_{11} } \hfill & {f_{12} } \hfill &\cdots \hfill & {f_{1j} } \hfill & \cdots \hfill & {f_{1n} } \hfill \\ {f_{21} } \hfill &{f_{22} } \hfill & \cdots \hfill & {f_{2j} } \hfill & \cdots \hfill & {f_{2n} } \hfill \\ { \vdots } \hfill & { \vdots } \hfill & \cdots \hfill & { \vdots } \hfill & \cdots \hfill & { \vdots } \hfill \\ {f_{i1} } \hfill & {f_{i2} } \hfill &\cdots \hfill & {f_{ij} } \hfill & \cdots \hfill & {f_{in} } \hfill \\ { \vdots } \hfill &{ \vdots } \hfill &\cdots \hfill & {\vdots } \hfill & \cdots \hfill & { \vdots } \hfill \\ {f_{J1} } \hfill & {f_{J2} } \hfill & \cdots \hfill & {f_{Jj} } \hfill &\cdots \hfill & {f_{Jn} } \hfill \\ \end{array} } \right] \hfill \\ \end{gathered}$$

where ${A}_{j}$ denotes the alternatives $j$, $j = 1,2, \ldots ,J:$ ${F}_{i}$ represents the $ith$ alternative or criterion, $i = 1,2, \ldots ,n,$ related to the $ith$ alternative; and ${f}_{ij}$ is a crips value indicating the performance rating of each alternative ${A}_{i}$ with respect to each criterion ${F}_{j}$.

*Step 2* Calculate the normalized decision matrix $\mathsf{R}\left(=\left[{r}_{ij}\right]\right).$ The normalized decision matrix ${r}_{ij}$ is computed as;

9 $$r_{ij} = \frac{{f_{ij} }}{{\sqrt {\mathop \sum \nolimits_{j = 1}^{n} f_{ij}^{2} } }}\quad = 1,2 \ldots ,J;\;I = 1,2, \ldots ,n.$$

*Step 3* Calculate the weighted normalized decision matrix by multiplying the normalized decision matrix by its associated weights. The normalized value ${v}_{ij}$ is calculated as:

10 $${v}_{ij}={w}_{i}*{r}_{ij},\; j=\text{1,2},\dots ,\;J,\; i=\text{1,2},\dots ,n,$$

where ${w}_{i}$ represents the weight of the $ith$ attribute or criterion.

*Step 4* Determine the positive-ideal and negative-ideal solutions

11 $$\begin{aligned} A^{*} & = \left\{ {v_{1}^{*} , v_{2,}^{*} \ldots .,v_{i}^{*} } \right\} \\ & = \left\{ {\left( {\mathop {\max }\limits_{j} v_{ij} {|}i \in {\rm I}^{\prime}} \right),\left( {\mathop {\min }\limits_{j} v_{ij} {|}i \in I^{\prime\prime}} \right)} \right\}, \\ \end{aligned}$$

12 $$\begin{aligned} A^{ - } & = \left\{ {v_{1}^{ - } , v_{2}^{ - } , \ldots ,v_{i}^{ - } } \right\} \\ & = \left\{ {\left( {\mathop {\min }\limits_{j} v_{ij} {|}i \in {\rm I}^{\prime}} \right),\left( {\mathop {\max }\limits_{j} v_{ij} {|}i \in I^{\prime\prime}} \right)} \right\}, \\ \end{aligned}$$

where ${\rm I}^{\prime}$ is associated with the benefit criteria and $I^{\prime\prime}$ is associated with the cost criteria.

*Step 5* Calculate the separate measures using the n-dimensional Euclidean distance. The separation of each alternative from the positive ideal solution is given as $({D}_{j}^{*})$ is given as

13 $$D_{j}^{*} = \sqrt {\mathop \sum \limits_{i = 1}^{n} \left( {v_{ij} - v_{i}^{*} } \right)^{2} } , \quad j = 1,2, \ldots ,J.$$

Similarly, the separation of each alternative from the negative ideal solution $D_{j}^{ - }$ is as follows.

14 $$D_{j}^{ - } = \sqrt {\mathop \sum \limits_{i = 1}^{n} \left( {v_{ij} - v_{i}^{ - } } \right)^{2} } , \quad j = 1,2, \ldots ,J.$$

*Step 6* Calculate the relative closeness of the ideal solution and rank the performance order. The relative closeness of the alternative $A_{j}$ can be expressed as

15 $$CC_{j}^{*} = \frac{{D_{j}^{ - } }}{{D_{j}^{*} + D_{j}^{ - } }},\quad j = 1,2, \ldots ,J,$$

where ${CC}_{j}^{*}$ index value lies between 0 and 1. The larger the index value the better the performance of the alternatives.

### ARAS

The ARAS model which has a unique advantage over other models is more specific and can assess the degree of performance of each alternative. This is achieved by comparing each alternative with an optimal alternative. The ARAS model, a multi-criteria evaluation method initially proposed by Zavadskas and Turskis^62^ and Zavadskas et al.^63^ is essentially used for optimally assessing, ranking, and appropriately placing the different alternatives in order of their significance within a given criterion. In a series of steps, the implementation of the ARAS model in the predictive process begins with the development of a decision matrix and then the generation of weighted criteria. The steps highlighted below are vital for the effective application of ARAS in decision-making and evaluation processes.

*Step 1* Generation of decision-making matrix (DMM). The DMM consists of $m$ feasible alternatives (rows) and rated on $n$ signfull criteria (columns).

16 $$x=\left[\begin{array}{ccc}\begin{array}{c}{x}_{01}\\ \vdots \\ \begin{array}{c}{x}_{i1}\\ \vdots \\ {x}_{m1}\end{array}\end{array}& \begin{array}{c}\cdots \\ \ddots \\ \begin{array}{c}\cdots \\ \ddots \\ \cdots \end{array}\end{array}& \begin{array}{cc}\begin{array}{c}{x}_{0j}\\ \vdots \\ \begin{array}{c}{x}_{ij}\\ \vdots \\ {x}_{mj}\end{array}\end{array}& \begin{array}{cc}\begin{array}{c}\cdots \\ \ddots \\ \begin{array}{c}\cdots \\ \ddots \\ \cdots \end{array}\end{array}& \begin{array}{c}{x}_{0n}\\ \vdots \\ \begin{array}{c}{x}_{in}\\ \vdots \\ {x}_{mn}\end{array}\end{array}\end{array}\end{array}\end{array}\right];\;\;i=\overline{0,m}, = \overline{1,n,}$$

where $m$—number of alternatives, $n$—number of criteria describing each alternative, ${x}_{ij}$—value representing the performance value of the $i$ alternative in terms of the $j$ criterion. ${x}_{oj}$—the optimal value of $j$ criterion.

If the optimal value of $j$ criterion is unknown, then

$${x}_{0j}= \underset{i}{\text{max}}{x}_{ij},\; if\;\underset{i}{\text{max}}{x}_{ij}$$ is preferable

$${x}_{0j}= \underset{i}{\text{min}}{x}_{ij}^{*}, \;if \;\underset{i}{\text{min}}{x}_{ij}$$ is preferable

*Step 2* in the second stage, the initial value of all the criteria is normalized—defining values ${\overline{x} }_{ij}$ of normalized decision matrix $\overline{x }$ is shown below.

17 $$\overline{x }=\left[\begin{array}{ccc}\begin{array}{c}{\overline{x} }_{01}\\ \vdots \\ \begin{array}{c}{\overline{x} }_{i1}\\ \vdots \\ {\overline{x} }_{m1}\end{array}\end{array}& \begin{array}{c}\cdots \\ \ddots \\ \begin{array}{c}\cdots \\ \ddots \\ \cdots \end{array}\end{array}& \begin{array}{cc}\begin{array}{c}{\overline{x} }_{0j}\\ \vdots \\ \begin{array}{c}{\overline{x} }_{ij}\\ \vdots \\ {\overline{x} }_{mj}\end{array}\end{array}& \begin{array}{cc}\begin{array}{c}\cdots \\ \ddots \\ \begin{array}{c}\cdots \\ \ddots \\ \cdots \end{array}\end{array}& \begin{array}{c}{\overline{x} }_{0n}\\ \vdots \\ \begin{array}{c}{\overline{x} }_{in}\\ \vdots \\ {\overline{x} }_{mn}\end{array}\end{array}\end{array}\end{array}\end{array}\right];\;\;i=\overline{0,m}, = \overline{1,n,}$$

The criteria whose preferable values are maxima are normalized as follows.

18 $${\overline{x} }_{ij}=\frac{{x}_{ij}}{\sum_{i=0}^{m}{x}_{ij}}$$

The criteria whose preferable values are minima are normalized by applying a two-stage procedure.

19 $${x}_{ij}=\frac{1}{{x}_{ij}^{*}} ; {\overline{x} }_{ij}=\frac{{x}_{ij}}{\sum_{i=0}^{m}{x}_{ij}}$$

When dimensionless values of the criteria are known, all criteria originally having different dimensions are compared.

*Step 3* Defining the normalized weighted matrix—$\widehat{x}$. It is possible to evaluate the criteria with weights $0<{w}_{j}<1.$ Only well-founded weights are used because weights are subjective and influence the solution. The values of the weights are usually determined by an expert method. The sum of the weights ${w}_{j}$ would be limited as follows.

20 $$\sum_{j=1}^{n}{w}_{j}=1$$

21 $$\overline{x }=\left[\begin{array}{ccc}\begin{array}{c}{\widehat{x}}_{01}\\ \vdots \\ \begin{array}{c}{\widehat{x}}_{i1}\\ \vdots \\ {\widehat{x}}_{m1}\end{array}\end{array}& \begin{array}{c}\cdots \\ \ddots \\ \begin{array}{c}\cdots \\ \ddots \\ \cdots \end{array}\end{array}& \begin{array}{cc}\begin{array}{c}{\widehat{x}}_{0j}\\ \vdots \\ \begin{array}{c}{\widehat{x}}_{ij}\\ \vdots \\ {\widehat{x}}_{mj}\end{array}\end{array}& \begin{array}{cc}\begin{array}{c}\cdots \\ \ddots \\ \begin{array}{c}\cdots \\ \ddots \\ \cdots \end{array}\end{array}& \begin{array}{c}{\widehat{x}}_{0n}\\ \vdots \\ \begin{array}{c}{\widehat{x}}_{in}\\ \vdots \\ {\widehat{x}}_{mn}\end{array}\end{array}\end{array}\end{array}\end{array}\right];\;\;i=\overline{0,m}, = \overline{1,n.}$$

Normalized weighted values of all criteria are calculated as follows

22 $$\hat{x}_{ij} = \overline{x}_{ij} w_{j} ;i = \overline{0,m} ,$$

where $w_{j}$ is the weight of the $j$ criterion and $\overline{x}_{ij}$ is the normalized rating of the $j$ criterion.

*Step 4* Determination of the optimality function as shown in Eq. (23).

23 $$S_{i} = \mathop \sum \limits_{j = 1}^{n} \hat{x}_{ij} ; i = \overline{0,m} ,$$

where ${S}_{i}$ is the value of the optimality function of $i$ alternative.

*Step 5* The degree of alternate utility is determined by a comparison of the variant, which is analysed with the ideally best one ${S}_{0}$. The equation used for calculating the utility degree ${K}_{i}$ of an alternative ${a}_{i}$ is shown in Eq. (24).

24 $$K_{i} = \frac{{S_{i} }}{{S_{0} }} ; i = \overline{0,m} .$$

where ${S}_{i}$ and ${S}_{0}$ are the optimality criterion obtained in Eq. (23).

25 $$SN=\frac{TP}{TP+FN}SP=\frac{TN}{TN+FP}$$

## Results

### Field evaluation

**Figure 4:**
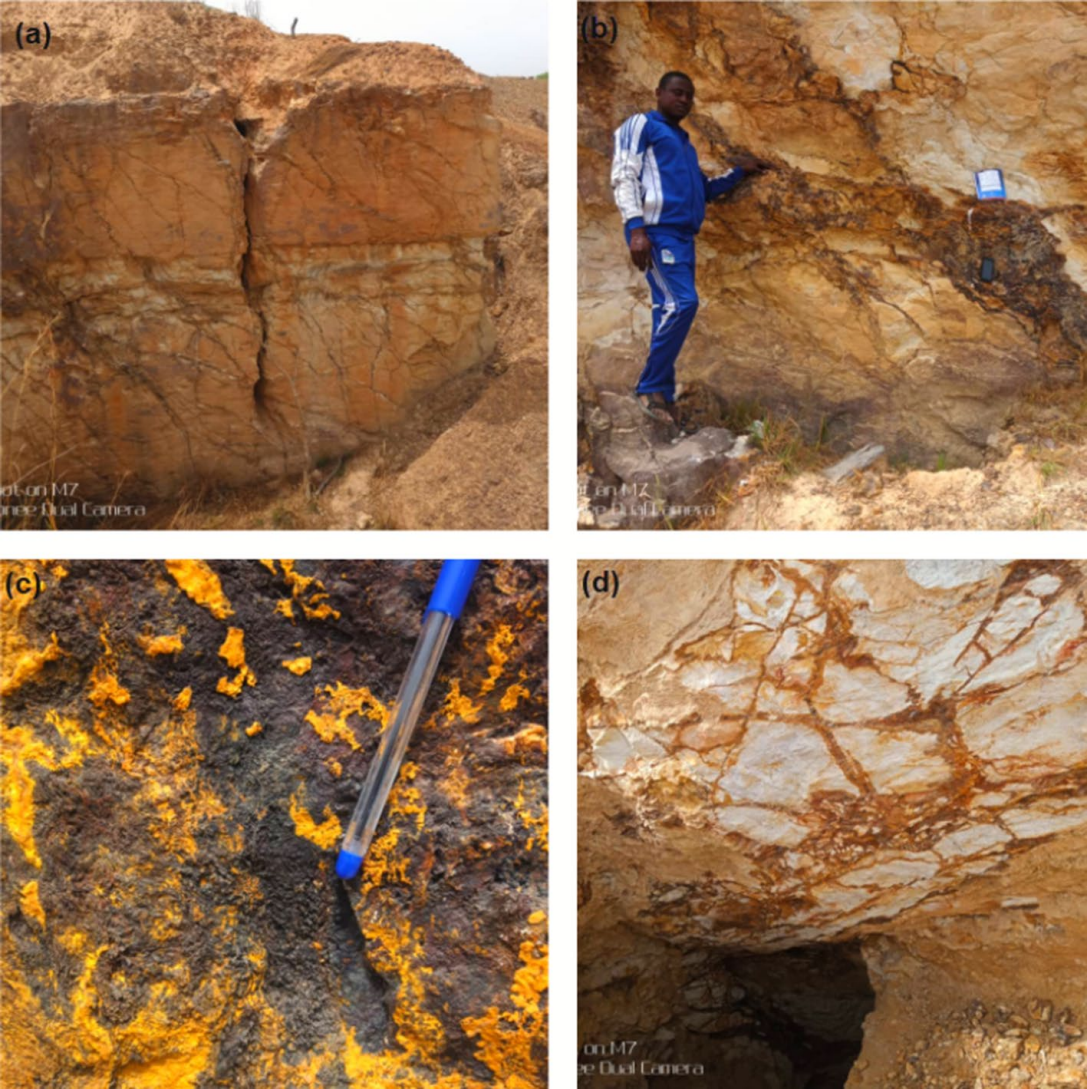
Field exposure of barite mineralisation within the Awe-Obi Area (**a**) Keana host rock (**b**) Barite occurrences within host rock (**c**) Limonitic alteration associated with barite mineralisation (**d**) Fracture intensity/brecciation associated with barite mineralisation. An informed consent was obtained for publication of identifiable images.

Evidence from field studies suggests that barite occurrences within the study location are hosted within the Cretaceous sediments comprising arkosic sandstones and siltstones of the Awe and Keana Formations (Fig. 4a). The barite-hosting lithological units appear to be poorly sorted and characterised by an interconnected network of rock fractures within which barite mineralisation occurs as infillings. Mesoscopic observations of vein mineralisation suggest the presence of numerous iron alteration minerals, including siderite and limonite, within a dominant clay-altered lithology (Fig. 4b and c). Mineral deposit bodies generally occur within zones of high lineament density, which may have served as channels for the migration of hydrothermal fluids, favouring the deposition of metallic and non-metallic minerals. Vein mineralisation is generally an open-space infilling of a series of steeply dipping fractures that occurs at the flanks of domain structures. Mineralised veins generally trend NW/SE and N/S, occurring as discordant bodies with a strike length of veins between 50–150 m and a width of a few centimetres to approximately 3.5 m. The veins have sharp contacts with the sandstone hosts and, in some instances, terminate abruptly and commonly split into narrow, few centimetre-wide veinlets. Vein margins were commonly brecciated, and the brecciated zones were similarly mineralised (Fig. 4d).

### Spatial data evaluation

**Figure 5:**
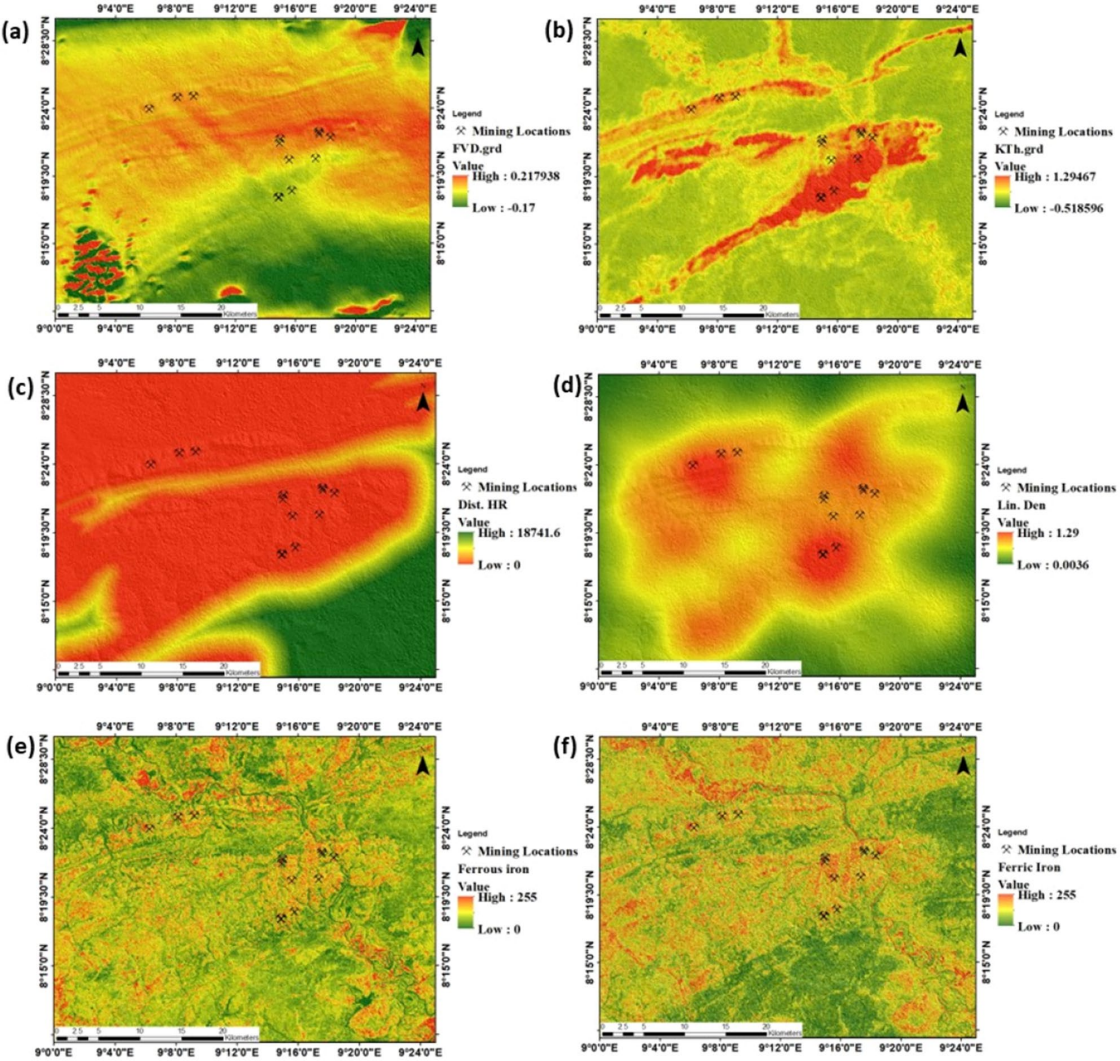
Evidential data for barite predictive modelling (**a**) FVD (**b**) K/Th ratio imagery (**c**) Distance to host rock (**d**) Lineament Density (**e**) Ferrous Iron alteration (**f**) Ferric Iron alteration.

Exploration of barite mineralisation using GIS data predictive modelling was facilitated by the spatial integration of geophysical, lithological, structural, and satellite data. The geophysical data used in this study included the FVD (Fig. 5a), K/Th ratio map (Fig. 5b), distance to the host rock (Fig. 5c), lineament density map (Fig. 5d), ferrous iron alteration map (Fig. 5e), and ferric iron alteration map (Fig. 5f). The magnetic variation within the FVD imagery ranges from − 0.173 nT to 0.21 nT with a mean value of 0.00023 nT and a standard deviation of 0.0087 nT. Analysis of the FVD imagery suggests a generally high magnetic signature within the central to eastern axes of the study location (Fig. 5a). The K/Th ratio is characterised by a minimum intensity of − 0.518, a maximum intensity of 1.287, a mean intensity of 0.081, and a standard deviation of 0.10. Zones with high K/Th intensities were more prominent within the central part of the study area (Fig. 5b). The lithological data used in this study included proximal distances to the host rock. Occurrences of sandstone formation were considered to be the host rock for barite mineralisation. The spatial data on Euclidean distances to host rock generated is defined by a maximum distance of 18.74 km, a minimum distance of 0 km, a mean distance of 17.23 km, and a standard deviation of 3.36 km. The analysis of this image suggests that sandstone formations occur mostly in the central and northern parts of the study area (Fig. 5c). Statistically, this formation accounts for 947.98 km2 of the study area.

Spatial information on geological structure consists of lineament intensity as shown in Fig. 5d. Lineament density within the study area is statistically characterised by a maximum and minimum intensity of 1.299 and 0.0036 respectively, a mean intensity of 0.57, and a standard deviation of 0.287. Zones of high lineament intensity were commonly observed within the central parts of the study location (Fig. 5d). Spatial information on ferrous iron alteration is categorised from low (green) to high (red). The low ferrous iron intensity is defined by a normalised digital number of 0, while the high ferrous iron intensity is represented by a normalised digital number of 255. Zones of high ferrous iron intensity are scattered throughout the study location but more prominent in the western, south-eastern, central, and northern parts of the study location (Fig. 5e). Information on ferric iron intensity is defined by a maximum of 255, a minimum of 0, a mean of 110.80 and a standard deviation of 60.87. Maximum and minimum ferric iron intensity were represented by colour variations from green (minimum) to red (maximum). High ferric iron alteration zones were observed within the western to the north-central, south-eastern, and northern axes of the study location (Fig. 5f).

### Variable correlation analysis

The statistical correlation used in this study included the Pearson correlation matrix and CAE. Table 2 illustrates the correlation matrix for the spatial data used in the study. Analysis of this table suggests a generally low to moderate correlation amongst predictive variables, However, a more significant positive correlation of 0.6 and 0.54 was observed between spatial data on ferric and ferrous iron alterations, lineament density, and K/Th ratio.

**Table 2:** Pearson correlation matrix for spatial data.

Variables	Ferric Iron	Ferrous Iron	Lin Den	KTh ratio	DHR	FVD
Ferric iron	1	0.610	0.073	0.149	− 0.103	0.128
Ferrous iron	0.610	1	0.165	0.067	0.003	0.048
Lin. Den	0.073	0.165	1	0.540	− 0.435	0.300
K/Th ratio	0.149	0.067	0.540	1	− 0.239	0.154
DHR	− 0.103	0.003	− 0.435	− 0.239	1	− 0.248
FVD	0.128	0.048	0.300	0.154	− 0.248	1

**Figure 6:**
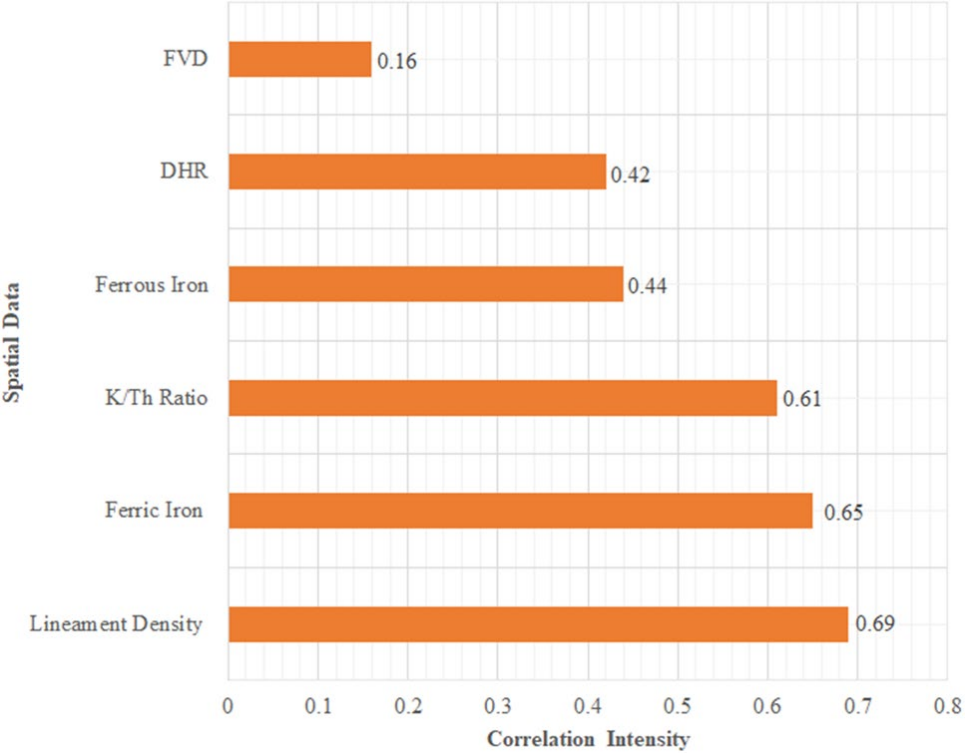
Showing the spatial relationship between the dependent and independent variables based on their correlation intensity.

The spatial relationship between the dependent and independent variables was assessed using CAE (Fig. 6). The evaluation of this analysis suggests that all the variables used in the study displayed a positive correlation with known barite occurrences. However, the maximum association was observed between spatial data on lineament density and ferric iron alteration, defined by a statistical association of 0.69 and 0.64 respectively. The minimum spatial association was observed for the proximal distances to sandstone (0.42) and the FVD image (0.16). Table 3 shows a statistical summary of the computed weights extracted from the spatial data correlation.

**Table 3:** Statistical summary of computed weights extracted from spatial data correlation.

S/No	Spatial data	Weights
1	FVD	0.16
2	DHR	0.42
3	Ferrous Iron	0.44
4	K/Th Ratio	0.61
5	Ferric Iron	0.65
6	Lineament Density	0.69

### Mineral potential mapping

**Figure 7:**
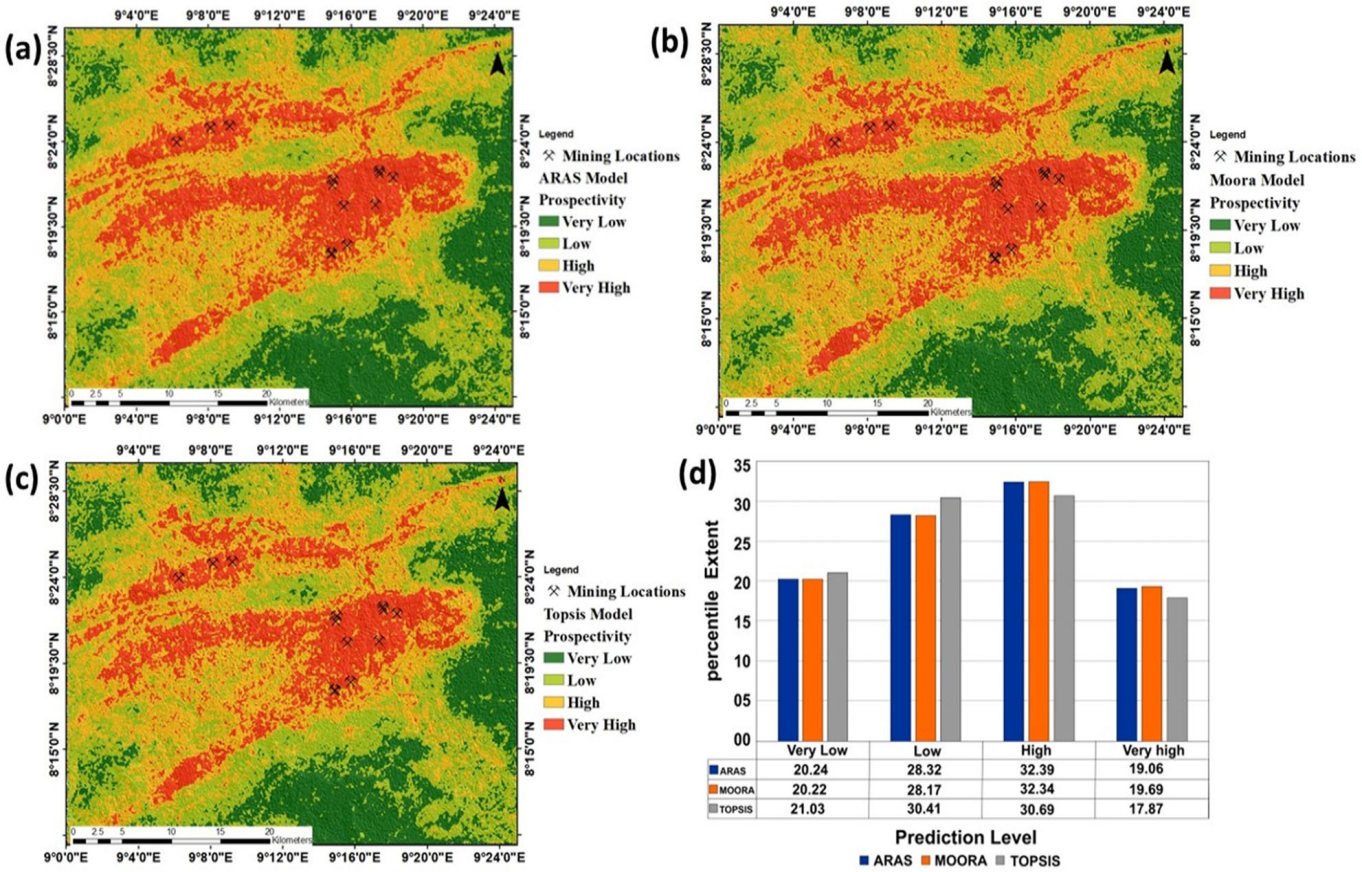
Predictive modelling and statistical quantification of predictive models (**a**) ARAS Predictive model, (**b**) MOORA Predictive model (**c**) TOPSIS Predictive model (**d**) Percentile estimation of prediction classes.

Data integration through the application of multi-criteria decision models (ARAS, MOORA, and TOPSIS) suggests that the central and north-central parts of the study location are the most favourable for barite exploration. Spatially, a high correlation exists among all the predictive maps generated using multi-criteria predictive models. However, the application of the ARAS model suggests prediction level varies from 0.25 to 1.0 with a mean value of 0.63 and a standard deviation of 0.169. The discretization of the ARAS predictive model into four classes suggests that the very low potential zone accounts for 20.23% which translates to 342.53 km^2^ of the study location (Fig. 7a). The very high potential class within this model covers an approximate area of 322.57 km^2^ which is equivalent to 19.05% of the study area. The spatial application of the MOORA model is characterised by prediction statistics ranging from 0.107 to 0.43 with a mean value of 0.27 and a standard deviation of 0.07. A discretisation of the resultant model into four classes shows that the very low potential class covers an approximate extent of 342.16 km^2^ accounting for 20.2% of the study location (Fig. 7b). The very high predictive class is defined by an area extent of 326.16 km^2^, accounting for 19.26% of the study area. Application of the TOPSIS model suggests that predictive levels vary from 0.027 to 0.844, with a mean of 0.43 and a standard deviation of 0.188. A discretisation of this model into four predictive classes shows that the very low predictive class covers an approximate area of 355.89 km^2^, which accounts for 21.028% of the study location. The very high predictive class covers an area extent of 302.42 km^2^, accounting for 17.86% of the study location (Fig. 7c). A percentile estimation of all the prediction classes is shown in Fig. 7d.

### Model validation

**Figure 8:**
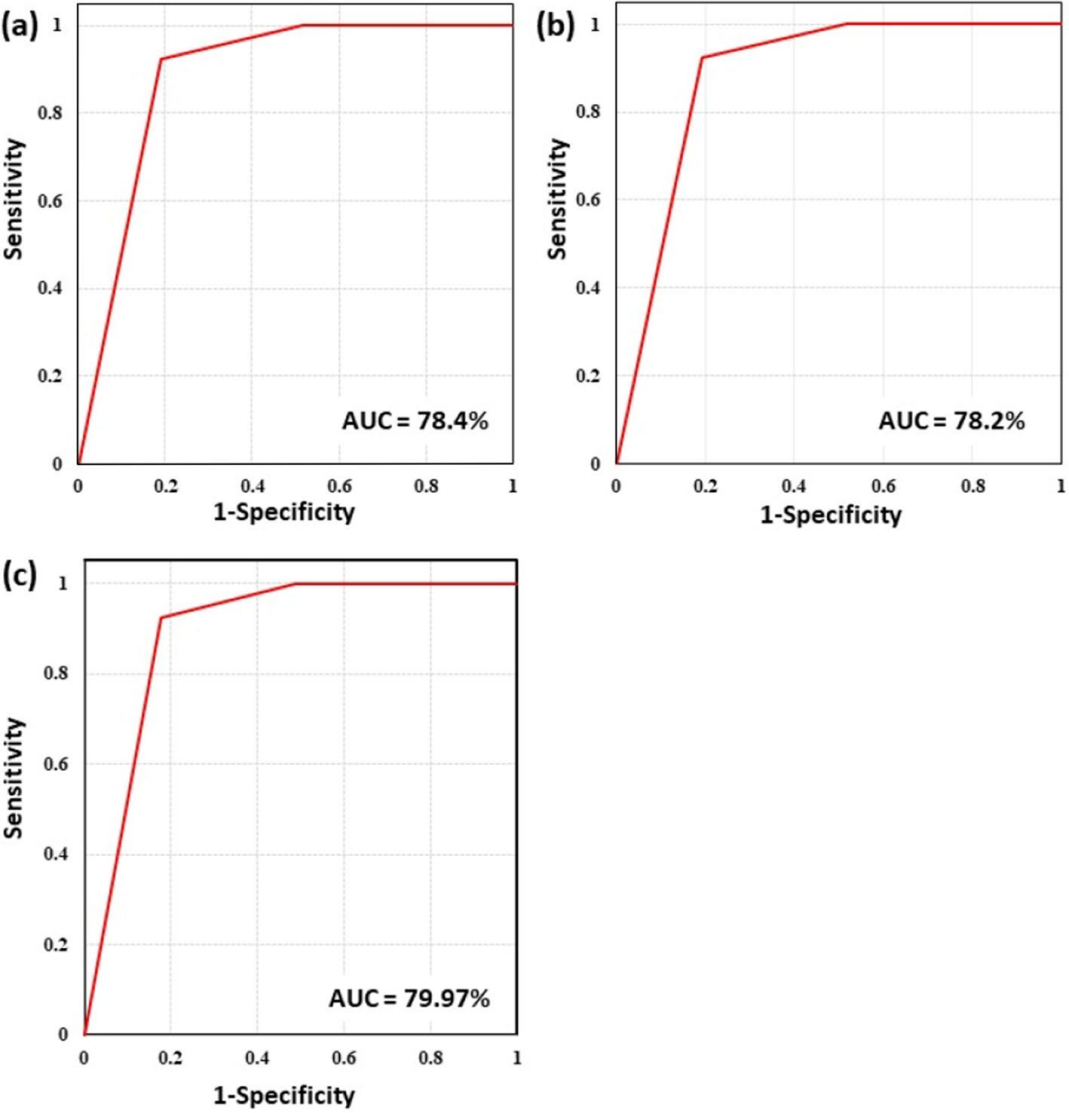
Statistical validation of predictive models using ROC/AUC (**a**) ARAS model (**b**) MOORA Model (**c**) TOPSIS Model.

A visual assessment of the spatial relationship between mineralisation and mineral predictive maps suggests a high proximal occurrence of known barite resources within zones of high and very high potential. Statistical assessment using the ROC technique revealed a preferential affinity of the ROC plot towards the top left axis of the graph, far above the marginal line. However, the AUC assessment for these graphs shows that the prediction levels were greater than 70%. The TOPSIS model exhibited the best prediction accuracy of 79.7% (Fig. 8c). This was closely followed by the ARAS model with a prediction accuracy of 78.4% (Fig. 8a). and the MOORA model with a prediction accuracy of 78.2% (Fig. 8b).

## Discussion

Barite deposits are known to occur extensively across the Nigerian BT and have been studied using diverse approaches including geochemical, structural, and geophysical tools^37,38,64,65^. Within the study location, barite mineralization is confined to the Keana sandstones and characterized by numerous geological, mineralogical, textural, and structural attributes, which facilitates their precise mapping using geospatial data. We report that within the MBT, barites are hosted predominantly by the sandstones of the Keana Formation. The proximal association of barite mineralization with the Keana sandstone formation can be attributed to the effective leaching of feldspar and the liberation of Ba from these sandstones by basinal fluids. We report that the formation of barites in MBT is consistent with the geochemical character of barium in its geochemical circle in which barium (Ba^2+^, ironic radius = 1.43A) is extensively substituted for potassium (K^+^, ionic radius = 1.33A) and thus, enriched in rocks carrying potassium feldspars, for instance, feldspathic and arkosic sandstones of Awe and Keana Formations. The migrating hydrothermal solutions perhaps extracted barium from the surrounding rocks (and enriched in relation to potassium) along fractures and lineament zones in the sedimentary sequence. The solutions were subsequently deposited as barites in favorable structures.

Structurally, field observations suggest that barite mineralization dissects the host rock both concordantly and discordantly and displays a strong affiliation to the NW–SE and N-S trending fractures. The strong association of barites occurrences within the NW–SE and N-S trending fractures has also been observed in parts of the Southern BT^66^. Within the Azara area of the MBT, the occurrences of three barite varieties (pinkish, whitish, and smoky) in proximal association with the NW–SE veins were identified. However, the whitish and smoky types were observed in close affiliation with the N-S trending veins. Generally, the NW–SE trending veins are orthogonal to the principal trend of the Nigerian BT, while the N-S are comparatively younger displaying a cross-cutting relationship. Iron alterations such as siderites and limonite in association with barite mineralization have been commonly observed within the entire BT of Nigeria and are considered to be products of supergene enrichment resulting in extensive lateralization of the overburden veins.

Spatially, predictive mapping for barite mineralization within the MBT is more favoured by the application of knowledge-driven predictive models due to the lack of sufficient evidence on barite mineral occurrences. However, for the systematic development of knowledge-driven barite predictive models, it is often essential to evaluate spatial data for interference associated with collinearity defects as a means of detecting data redundancy. Although different approaches have been successfully applied for collinearity assessment, the application of the Pearson correlation matrix used in this study shows that the correlation among spatial data was less than the recommended threshold levels necessary to cause a collinearity effect. A precise understanding of the spatial relationship between the evidential data and known barite mineralization is fundamental for developing highly accurate predictive maps for barite mineralization. A statistical test using attribute correlation evaluation analysis exhibited a positive relationship between the predictive variables and known barite occurrences. The maximum spatial association with barite mineralization was observed from evidential data on lineament density, ferric iron alteration, and K/Th ratio. The relationship between geological structures and mineral deposit occurrence can be explained in terms of the migration of mineralizing fluids across specific geological formations. Zones of high fracture intensity would imply higher mobility of mineralizing fluids, and thus a substantial deposition of mineralization. The mobility of hydrothermal fluids within most geological media is often accompanied by significant alterations in the chemistry and mineralogy of rocks. Previous studies have often reported a proximal association between mineral deposits and zones of high fracture intensity^67^. In Nigerian BT, this association has been validated by^68^.

Within the MBT, evidence from field studies suggests barite mineralization occurs in proximity to iron and clay alterations and the contacts of barite veins are highly brecciated and occur in proximal association with siderite which is further oxidized to limonite and can be mapped on spatial data as ferrous and ferric iron alterations. Also, ASTER mapping of barite mineralization within the southern part of the Nigerian BT has revealed an intrinsic relationship between barite occurrences and zones of ferric and ferrous iron alterations.

Because barite mineralization across the BT is characterized by hydrothermal alterations, the use of the K/Th image showed the spatial extent of this alteration across the study location. Zones of high K/Th values indicated enrichment in K concentration typical of hydrothermal alteration zones. Primarily, a few lithological units are known to play host to barite mineralization across the Nigerian BT^38^. Barite occurrences within the study location are hosted by Turonian sandstones of the Keana Formation representing a late-stage mineralization episode. Thus, the presence of this litho-unit may be a reliable indicator for barite occurrence across the study location. Magnetic anomalies are considered reliable evidence for the presence of ore deposits or mineralization, although their concentration within mineralized systems may vary based on specific geological factors^69^. However, a positive anomaly associated with sedimentary rocks may be related to mineralization in the high lineament density zones due to the upward migration of hydrothermal fluids along fracture and fault planes. In the Nigerian BT, barite mineralization occurs in proximity to numerous ores including galena, sphalerite, and pyrite. The presence of these ores within most barite veins facilitates their mapping using magnetic data.

In the predictive mapping of mineral resources, the use of multi-criteria decision models has been credited with a substantial degree of accuracy and has been recommended as a viable exploration tool^20,70–72^. In this study, high accuracy levels in the prediction of barite occurrence were validated using the ARAS, MOORA, and TOPSIS models. However, the TOPSIS model with a prediction accuracy of 79.9% outperformed the accuracies of the MOORA (78.4%) and ARAS (78.2%) models. The difference in accuracy levels observed from these models can be attributed to complexities in the assignment of performance values to different alternatives within every evidential data. The multi-criteria decision models offer a defined solution to a given problem using different weighting systems which are developed by implementing diverse configurations on weighted values, scales, and normalization. Also, some multi-criteria decision models may attempt to present additional parameters which may affect the performance level of the resultant model. The high reliability of the TOPSIS model in comparison to the ARAS and MOORA models has been previously validated by Tende et al.^20^ in the predictive mapping of gold deposits in northern Nigeria. Unlike the MOORA and ARAS models, a high-performance level for the TOPSIS model is attributed to its ability to generate weights for various alternatives by presenting a scalar parameter that integrates the best and worst alternative measures in deducing the relative performance for every alternative. According to Hosmer and Lemeshow^73^, accuracy levels with AUC values above 70% are considered substantial and acceptable for statistical prediction.

## Conclusions

Multi-criteria decision models are fundamental tools for regionally exploring mineral deposit occurrences and have been successfully employed in the study of barite prospecting. Based on spatial data integration, high prospects for barite mineralization were identified within the central and north-central parts of the study location. These zones of high prospects are highly recommended for detailed exploration studies. In all the models applied in this study, a high degree of consistency existed in identifying barite prospective zones. The spatial consistency associated with these models is considered a fundamental attribute for ascertaining the primary reliability of multi-criteria models in exploration studies. Application of the ROC/AUC analysis further validated the reliability of the multi-criteria tool in GIS mapping and authenticated its implementation in exploration studies both within the MBT and other terrains with similar geologic characteristics. For future studies on mapping potential zones of barite mineralization, we recommend using the gravity method. This method takes advantage of the contrasting density of barite in the surrounding rocks. Additionally, other methods such as induced polarization, self-potential, and electrical resistivity to map barite and its accessory sulphide minerals are also recommended.

## Data Availability

Correspondence and requests for materials should be addressed to M.D.A. Data sets generated during the current study are available from the corresponding author on reasonable request.
